# COPING WITH COGNITIVE DECLINE AMONG OLDER ADULTS WITH MILD COGNITIVE IMPAIRMENT OR MILD DEMENTIA: A SCOPING REVIEW

**DOI:** 10.1093/geroni/igac059.2272

**Published:** 2022-12-20

**Authors:** Youngmin Cho, Donruedee Kamkhoad, Anna Beeber

**Affiliations:** University of North Carolina - Chapel Hill, Chapel Hill, North Carolina, United States; The University of North Carolina at Chapel Hill School of Nursing, Chapel Hill, North Carolina, United States; Johns Hopkins University, Baltimore, Maryland, United States

## Abstract

As cognitive impairment progresses, Persons with Mild Cognitive Impairment or Mild Dementia (PwMCI or MD) have trouble in adapting to changes in their cognitive functioning and, as a result, develop challenges in daily activities. If these challenges are not appropriately addressed, PwMCI or MD can experience poor health outcomes and quality of life. Coping, behavioral and cognitive efforts to regulate the distress from a certain stressful situation, is widely regarded as a fundamental determinant of health outcomes. We conducted a scoping review to understand what coping strategies PwMCI or MD used, and facilitators and barriers of the use of these coping strategies. Using the PRISMA-ScR guideline, we reviewed peer-reviewed empirical studies exploring coping experiences for cognitive impairment among community-dwelling PwMCI or MD. We used thematic synthesis to generate themes relevant to the coping strategies and the facilitators and barriers. Of 1267 studies identified, 12 qualitative studies were reviewed. Under three dimensions of coping strategy (i.e., problem-solving, emotional, and maladaptive coping strategies), six themes were identified: independent coping (e.g., use of reminder and practice of cognitive activities), collaborative/dependent coping (e.g., asking help as needed), reframing, expression of unpleasant feelings, comparing self to others, and avoidance. The themes for facilitators and barriers were social response to one’s cognitive impairment, assistance from informal care partners, and support from professionals. These findings can provide not only a better understanding of how people cope with their cognitive impairment but can also provide rationale for developing interventions to maximize the use of coping strategies.

